# Supplementary material for: Role of Exosomes in Immunotherapy of Hepatocellular Carcinoma
Source: Cancers (Basel). 2022 Aug 21;14(16):4036. doi: 10.3390/cancers14164036 (PMC9406927; doi:10.3390/cancers14164036)
Supplement: Supplementary file 1 [file cancers-14-04036-s001.zip › cancers-1784171-supplementary.pdf]

Table S1. Influence of exosomes on cells in tumor microenvironment of HCC

Production cell	Exosomes	Affected cell	Role	Reference
HCC cells	14-3-3ζ protein	Tumor-infiltrating T lymphocytes	Decrease the activation and proliferation of naïve T cells and deviate the differentiation of the latter from effector T cells to Tregs	[30]
	miR-15a-5p	CD8+ T cells	Inhibit PD-1 expression and suppress the development of HCC	[31]
	PCED1B-AS1	Receipt T cells, macrophages and receipt HCC cells	Regulate PD-Ls expression in receipt HCC cells and inhibit receipt T cells and macrophages	[32]
	circGSE1	Tregs	Facilitate HCC progression via inducing the expansion of Tregs	[33]
	HBV nucleic acids	Macrophages	Induce NKG2D expression in macrophages and further promote NK cells activation	[34]
	miR-92b	NK cells	Downregulate CD69 and NK cell-mediated cytotoxicity	[35]
	circUHRF1	NK cells	Inhibit NK cells function by upregulating the expression of TIM-3 via degradation of miR-449c-5p and inhibit NK cell-derived IFN-γ and TNF-α secretion	[36]
	TEXs	DC cells	Carry HCC antigens and trigger a strong DC-mediated immune response	[37]
	TEXs	DC cells	Activate DC cells and further promote T cells proliferation	[40]
	PD-L1	TAMs	GOLM1 promote PD-L1 stabilization and transport PD-L1 into TAMs with exosomes, further suppress CD8+ T cells	[41]
	miR-23a-3p	Macrophages	Upregulate PD-L1 expression in macrophages and further inhibit T cells function	[42]
	LOXL4	Macrophages	Upregulate PD-L1 expression in macrophages	[43]
	circTMEM181	Macrophages	Upregulate CD39 expression in macrophages, produce more adenosine and further impair CD8+ T cells function	[44]
	miR-99b	Macrophages	Re-educate TAMs toward antitumor phenotype (promote M1 while suppress M2 macrophage polarization)	[45]
	(Low level) hsa_circ_0074854	Macrophages	Exosomes with downregulate hsa_circ_0074854 can inhibit macrophage M2 polarization	[49]
	HMMR-AS1	Macrophages	Promote the M2 polarization of macrophages	[46]
	miR-146a-5p	Macrophages	Promote the M2 polarization of macrophages	[47]
	DLX6-AS1	Macrophages	Promote the M2 polarization of macrophages	[48]
	HMGB1	B cells	Activate B cells and promote TIM-1+ regulatory B cells expansion	[58]
	TEXs	Hepatocytes	Mobilize normal hepatocytes	[70]

	TEXs (adenylyl cyclase-associated protein 1)	HCC cells	Regulate the motile ability of HCC cells	[71]
	TEXs	Vascular endothelial cells	Promote angiogenesis	[62,63]
	CXCR4	Lymphatic endothelial cells	Promote lymph angiogenesis	[64]
	miR-92a-3p	Epithelia cells	Promote epithelial-mesenchymal transition and metastasis	[72]
	circ-0004277	Peripheral cells	Promote epithelial-mesenchymal transition and metastasis	[73]
DC cells	DEXs	T cells	Stimulate naïve T cells proliferation and induce T cells activation to become antigen-specific cytotoxic T lymphocytes	[39]
Macrophages	miRNAs (miR-223)	HCC cells	Prevent proliferation of HCC	[53]
	hsa_circ_0004658	HCC cells	Inhibit HCC progression	[57]
M2 macrophages	miR-660-5p	HCC cells	Promote HCC development and epithelial-mesenchymal transition	[50]
	miR-21-5p	CD8+ T cells	Facilitate CD8+ T cells exhaustion	[51]
	miR-27a-3p	HCC cells	Promote cancer stemness of HCC through down-regulating TXNIP	[52]
	(Lower level) miR-125a/b	HCC cells	Promote cell proliferation and stem cell properties of HCC cell by downregulation of CD90	[54]
	miR-92a-2-5p	HCC cells	Increase the invasion capacity of HCC	[56]
M1 macrophages	miR-326	HCC cells	Suppress proliferation, migration, invasion and advance apoptosis of HCC	[55]
Cancer-associated fibroblasts	circZFR	HCC cells	Promote HCC development	[65]
	TUG1	HCC cells	Promote migration, invasion and glycolysis	[66]
	miR-29b	HCC cells	Inhibit migration and invasion	[67]
Adipocytes	miR-23a/b	HCC cells	Promote HCC cell growth and migration	[68]
	circRNAs	HCC cells	Promote tumor growth and reduce DNA damage	[69]
Mesenchymal stem cells	miR-127-3p	Cancer stem cells	Block malignant behaviors of HCC-sourced cancer stem cells	[74]
	miR-15a	HCC cells	Downregulate SALL4 expression and thereby retard HCC development	[75]

HCC, hepatocellular carcinoma; PD-1, programmed cell death protein 1; NK, nature killer; DC, dendritic; PD-L1, programmed cell death ligand 1; TEX, tumor cell-derived exosomes; DEXs, DC-derived exosomes; TAMs, tumor-associated macrophages; Tregs, regulatory T cells.
